# Diagnostic Accuracy of PET with Different Radiotracers versus Bone Scintigraphy for Detecting Bone Metastases of Breast Cancer: A Systematic Review and a Meta-Analysis

**DOI:** 10.3390/jimaging9120274

**Published:** 2023-12-08

**Authors:** Maryam Zamanian, Giorgio Treglia, Iraj Abedi

**Affiliations:** 1 Department of Medical Physics, School of Medicine, Isfahan University of Medical Sciences, Isfahan 8174673461, Iran; m.zamanian@resident.mui.ac.ir (M.Z.); i.abedi@med.mui.ac.ir (I.A.); 2 Faculty of Biomedical Sciences, Università della Svizzera Italiana, 6900 Lugano, Switzerland; 3 Division of Nuclear Medicine and Molecular Imaging, Imaging Institute of Southern Switzerland, Ente Ospedaliero Cantonale, 6500 Bellinzona, Switzerland; 4 Faculty of Biology and Medicine, University of Lausanne, 1015 Lausanne, Switzerland

**Keywords:** bone scintigraphy, bone metastases, breast cancer, [^18^F]FDG, Na[^18^F]F, PET/CT, PET/MRI, PET

## Abstract

Due to the importance of correct and timely diagnosis of bone metastases in advanced breast cancer (BrC), we performed a meta-analysis evaluating the diagnostic accuracy of [^18^F]FDG, or Na[^18^F]F PET, PET(/CT), and (/MRI) versus [^99m^Tc]Tc-diphosphonates bone scintigraphy (BS). The PubMed, Embase, Scopus, and Scholar electronic databases were searched. The results of the selected studies were analyzed using pooled sensitivity and specificity, diagnostic odds ratio (DOR), positive–negative likelihood ratio (LR^+^–LR^−^), and summary receiver–operating characteristic (SROC) curves. Eleven studies including 753 BrC patients were included in the meta-analysis. The patient-based pooled values of sensitivity, specificity, and area under the SROC curve (AUC) for BS (with 95% confidence interval values) were 90% (86–93), 91% (87–94), and 0.93, respectively. These indices for [^18^F]FDG PET(/CT) were 92% (88–95), 99% (96–100), and 0.99, respectively, and for Na[^18^F]F PET(/CT) were 96% (90–99), 81% (72–88), and 0.99, respectively. BS has good diagnostic performance in detecting BrC bone metastases. However, due to the higher and balanced sensitivity and specificity of [^18^F]FDG PET(/CT) compared to BS and Na[^18^F]F PET(/CT), and its advantage in evaluating extra-skeletal lesions, [^18^F]FDG PET(/CT) should be the preferred multimodal imaging method for evaluating bone metastases of BrC, if available.

## 1. Introduction

Breast cancer (BrC) is the most common cancer among women. Based on IARC (International Agency for Research on Cancer) reports from 185 countries, 2.3 million new cases and a mortality rate of 6.9% have been reported. Furthermore, its prevalence is increasing due to lifestyle changes [1]. Despite many advances in the field of treatment methods such as surgery, radiotherapy, and hormone therapy, BrC recurrence and metastases to other organs are frequent, so this type of malignancy can be considered a systemic disease [2,3].

Bone is the most common site of distant metastases of BrC, which affects 8% of patients in the early stages and 85% in the advanced stages [4]. Bone metastases (BMs) first originate from the red bone marrow and subsequently cause structural changes classified into different metastatic lesions (osteolytic, osteosclerotic, or mixed lesions) [5].

Sometimes BMs cause pain in the spine and lower back with compression of the spinal cord, which affects the patient’s quality of life and increases the possibility of pathological fractures [6]. Therefore, early diagnosis of BMs can delay the progression of the disease and be useful for improving the quality of life of BrC patients, allowing them to choose appropriate treatments resulting in better outcomes [7].

In current imaging protocols, bone scintigraphy (BS) including planar and tomographic acquisitions such as Single Photon Emission Computed Tomography (SPECT) and computed tomography (CT) are used for the early detection of BMs and visceral metastases, respectively [8]. Magnetic Resonance Imaging (MRI) is another anatomy-based method that can be considered in this regard [9].

BS using radiolabeled diphosphonates may assist in assessing osteoblastic activity, enabling the visualization of bone turnover in the sites of BMs. Different Technetium-99m (^99m^Tc)-labeled diphosphonates are available, including Methylene 3-Diphosphonate-1, 2-Propanodicarboxylic acid (MDP—three different kits), Disphosphono-1,2-Propanodicarboxylic acid (DPD—two types), and Hydroxy Diphosphonate (HDP) [10]. However, the uptake of radiolabeled diphosphonates in BS may be due not only to BMs but also to bone fracture, arthritis, and infection, challenging the correct diagnosis of BMs [11]. CT scanning of bone tissue with high contrast can facilitate the detection of benign and malignant lesions, but the high dose to the patient and the limitation of the field of view (FOV) can be some of the disadvantages of this method [12]. Combining morphological and functional information through SPECT/CT hybrid imaging may provide better results in terms of diagnostic accuracy of BMs [13].

**Figure 1:**
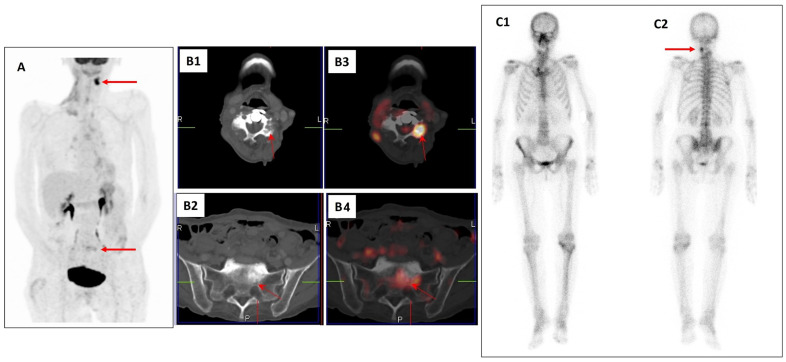
An example of a 77 year-old patient with a history of breast cancer restaged with [^18^F]FDG PET/CT (**A**,**B1**–**B4**) and bone scintigraphy with [^99m^Tc]Tc-diphosphonates (**C1**,**C2**). [^18^F]FDG PET (**A**), axial CT (**B1**,**B2**), and PET/CT images (**B3**,**B4**) detected morphological abnormalities with increased radiotracer uptake in the cervical spine and sacrum (arrows) suspicious for bone metastases (L = left, R = right, and P = posterior side). Bone scintigraphy of the same patient in anterior (**C1**) and posterior (**C2**) views also detected a focal area of increased radiopharmaceutical uptake in the cervical spine corresponding to the PET/CT finding (arrow). Biopsy confirmed bone metastases in the cervical spine and sacrum.

Another method with high diagnostic accuracy in oncology is positron emission tomography (PET) [13]. The most used PET radiotracers for the evaluation of BMs in BrC include the glucose analogue 2-deoxy-2-(^18^F)-fluoro-D-glucose ([^18^F]FDG) and Sodium [^18^F]Fluoride (Na[^18^F]F), which have different uptake mechanisms based on the glucose metabolism and bone turnover, respectively [14,15]. Recently, the use of hybrid PET/CT and PET/MRI imaging combining morphological and functional information has enhanced the diagnosis of BMs [16,17,18]. An example of bone metastases of BrC detected by [^18^F]FDG PET/CT and bone scintigraphy with [^99m^Tc]Tc-diphosphonates is shown in Figure 1.

Advances in equipment and radiotracers for diagnosing abnormalities make it necessary to update related protocols. Therefore, the current study was carried out to provide a comprehensive evaluation and comparison of the diagnostic accuracy of different nuclear medicine imaging methods for diagnosing BMs in BrC patients.

## 2. Materials and Methods

Our goal was to compare the diagnostic accuracy of different nuclear medicine imaging methods (BS, and PET with [^18^F]FDG and Na[^18^F]F) and the impact of anatomical modalities (CT and MRI) on the diagnosis of BM caused by BrC through a systematic review and meta-analysis. The review protocol has been registered on the Prospective Register of Systematic Reviews (PROSPERO) website (CRD42022379247). The systematic review and meta-analysis were written according to the 2020 PRISMA statement [19], and a written review protocol was drafted (Supplementary Table S1).

### 2.1. Sources and Strategy for the Literature Search

The electronic databases MEDLINE (PubMed), Embase (Elsevier and Ovid), and Scopus were evaluated. The Scholar database was examined to evaluate and avoid missing preprinted and arXiv articles and other articles. The following search string combining different keywords was used for the literature search: (“PET” OR “FDG” OR “fluorodeoxyglucose” OR “FDG-PET” OR “NaF” OR “sodium fluoride” OR “PET/CT” OR “PET/MR” OR “positron emission tomography” OR “bone scintigraphy” OR “bone scan”) AND (“breast cancer” OR “breast carcinoma” OR “breast neoplasm”) AND (“bone metastasis” OR “bone metastases” OR “recurrence”) AND (“sensitivity” OR “specificity” OR “accuracy” OR “diagnosis” OR “detection”). Additionally, references from the selected studies were checked to find additional eligible articles. The literature search, the study selection, the data extraction, and the quality assessment were performed by two reviewers independently. Regarding disagreements among the reviewers, agreements were reached in a consensus meeting.

The time range for the literature search using all databases was between 2010 and June 2023. The search results in the Embase database included Quick limit (“Humans”) and publication type (“Article”, “Article in Press”, and “Preprint”) items. In addition, the search in the PubMed database included “Humans”, and the search in the Scopus database about “Article title, Abstract, Keywords” included document type “Article”, source type “journal”, and the keyword “Humans”.

### 2.2. Research Question and Study Exclusion Criteria

The PICO question was: what is the diagnostic performance, in terms of sensitivity and specificity (outcome), of PET with [^18^F]FDG or Na[^18^F]F combined with CT or MRI (intervention) versus BS (control) in the evaluation of BMs of BrC patients (participants)?

Original articles that were compliant to this research question were included.

Taking into consideration the research question, the exclusion criteria were: (a) all studies that were reviews, case reports, editorials, letters, notes, and conference abstracts in the field of interest; (b) studies that lacked information on the desired effect size (sensitivity and specificity); (c) studies that were outside the field of interest of this review (for instance studies that evaluated BMs of other cancers beyond BrC with the selected index test).

### 2.3. Risk of Bias and Publication Bias Assessment

The Quality Assessment of Diagnosis Accuracy Studies (QUADAS-2) tool, which is specific for examining diagnostic studies, was used to evaluate the quality of the selected studies. This tool includes questions about four domains: patients (setting, intended use of index test, presentation, prior testing), index test(s), reference standard, and flow and timing. The possible answers to each question were: yes, no, or unclear.

To evaluate publication bias, the interpretation of the funnel plot was used.

### 2.4. Data Extraction

Results of each imaging modality among ([^18^F]FDG-Na[^18^F]F) PET, PET(/CT, or /MRI, and [^99m^Tc]Tc-diphosphonates BS to detect BMs caused by BrC were extracted, including the number of true positives (TPs), false positives (FPs), false negatives (FNs), and true negatives (TNs) from a patient- and lesion-based analysis. Other descriptive information extracted from the studies included “authors and year of article publication”, “country”, “patient age”, “sample size”, “type of study (retrospective-prospective)”, “metastasis site”, “the type of PET radiotracer ([^18^F]FDG-Na[^18^F]F)”, “type of diphosphonates (MDP-DPD-HDP)”, “ BS imaging acquisition (planar-tomographic)”, and “devices model”. In addition, some index test characteristics were extracted, including “injected dose”, “waiting time for imaging”, “the number of bed positions”, “time per bed position”, “most frequent type of bone metastases”, and “frequent sites of bone metastases”. Lastly, the reference standard used to validate imaging findings in the included studies was extracted.

### 2.5. Statistical Analysis

All analyses were performed using Microsoft Excel (Microsoft 2016, Microsoft, Seattle, WA, USA), Meta-DiSc software (Version 1.4, Spain), and Stata software (Version 17, College Station, TX, USA). The pooled sensitivity, specificity, diagnostic odds ratio (DOR), positive and negative likelihood ratio (LR^+^–LR^−^), and summary receiver–operating characteristic (SROC) curves were analyzed for each imaging modality. The pooled analysis was performed with the corresponding 95% confidence interval (CI) using a random-effects (DerSimonian and Laird) model. The degree of heterogeneity was estimated by *I*^2^ statistics (significant heterogeneity for *I*^2^ > 70%), and regression analysis was used to find factors causing heterogeneity.

## 3. Results

### 3.1. Literature Search and Selection of Studies

The number of studies found in each database were 1953 for Embase, 272 for PubMed, 4046 for Scopus, and 7440 for Scholar. Thus, the total number of extracted articles in the initial search was 13,711, and after removing 6738 duplicate studies, 6973 studies remained.

**Figure 2:**
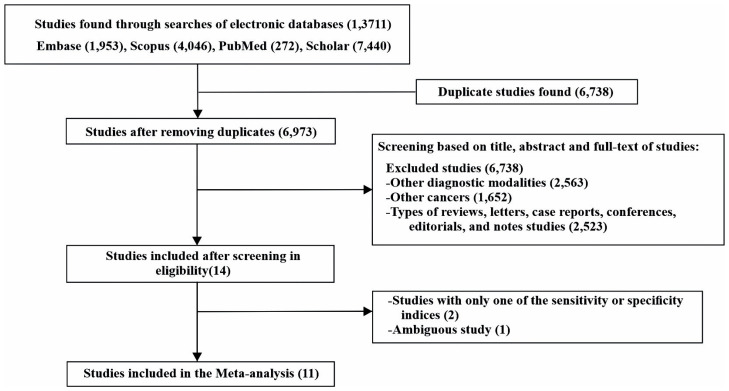
Flowchart illustrating the selection of studies comparing the diagnostic accuracy of PET, PET(/CT, or /MRI) with [^18^F]FDG or Na[^18^F]F versus [^99m^Tc]Tc-diphosphonates bone scintigraphy to detect bone metastases from breast cancer.

After removing further studies based on the exclusion criteria, 13 studies were included in the systematic review and 11 studies were included in the meta-analysis (Figure 2).

### 3.2. Results of the Risk of Bias Assessment

**Figure 3:**
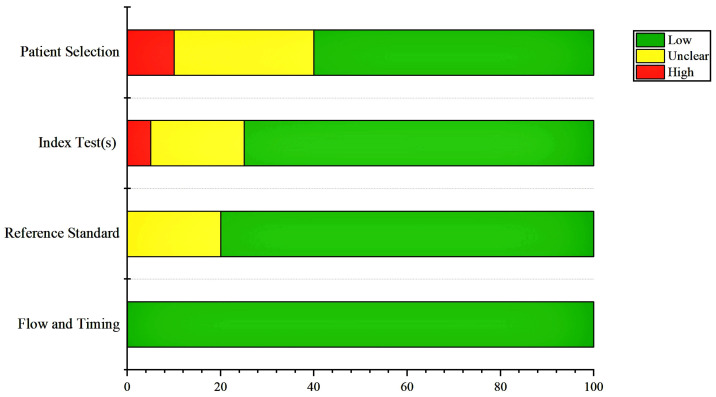
The results of the risk of bias assessment using the QUADAS-2 tool for each of the four domains assessed. The high, low, and unclear risk of bias percentages are marked with red, green, and yellow colors, respectively.

The quality of the studies included in this study was evaluated using the QUADAS-2 tool, and the results of the evaluation of the methodology showed that the risk of bias was very low for most parts of the studies (Figure 3).

### 3.3. Study Characteristics

A total of 753 BrC patients with a mean age of 57.7 years from 11 studies were included in the meta-analysis evaluation. The studies included in the systematic review were prospective (six studies) or retrospective (seven studies).

Diagnostic modalities included BS performed using planar or tomographic (SPECT or SPECT/CT) acquisitions, [^18^F]FDG PET, and [^18^F]FDG or Na[^18^F]F PET/CT.

The field of view in some studies was limited to middle or proximal femurs, whereas in other studies the full skeleton was imaged.

Radiolabeled PET tracers used in the included studies included [^18^F]FDG (eight articles) and Na[^18^F]F (four articles). Radiolabeled diphosphonates used for BS included MDP (three studies), HDP (one study), and DPD (two studies).

The algorithm of all systems was an iterative reconstruction and the scan time for BS ranged between 20 min and 3 h after tracer injection; the injected activity of radiolabeled diphosphonates ranged from 55 to 1000 MBq. The scan time for PET and combined scans (PET/MRI and PET/CT) ranged from 45 min to 2 h after radiotracer injection; the PET radiotracer activity injected ranged between 184 and 550 MBq. The number of bed positions for PET scan studies was 3–12 and the time was approximately 2.5–4 min per bed position. The follow up time ranged between 3.8 and 33 months.

Some studies, in addition to the diagnostic methods considered, had evaluated other diagnostic methods which were excluded from this evaluation. The descriptive characteristics of the included studies and patients are presented in Table 1 and Table 2. The results in terms of diagnostic performance of the index test and comparison are illustrated in Table 3.

### 3.4. Evaluation of the Diagnostic Accuracy of Imaging Modalities

**Figure 4:**
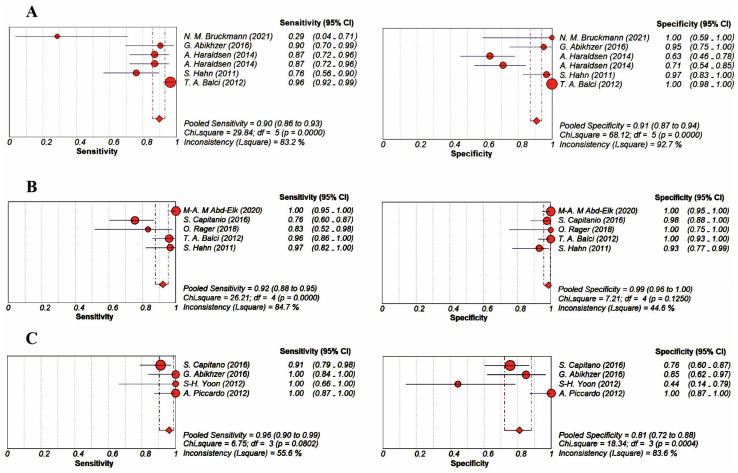
Forest plots for the patient-based pooled sensitivity and specificity of bone scans with [^99m^Tc]Tc-diphosphonates [20,25,26,31,32] (**A**), [^18^F]FDG PET/CT [22,23,24,31,32] (**B**), and Na[^18^F]F PET/CT [24,25,29,30] (**C**). The dotted red lines indicate the range of pooled sensitivity and specificity values.

**Figure 5:**
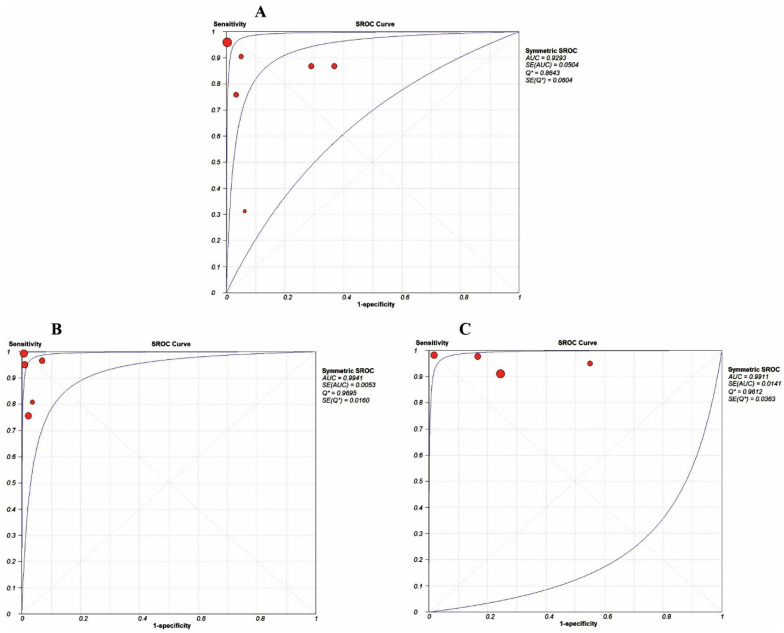
SROC curves for BS with [^99m^Tc]Tc-diphosphonates (**A**), [^18^F]FDG PET/CT (**B**), and Na[^18^F]F PET/CT (**C**). The blue lines show the 95% confidence interval value (95% CI) and the red points show the values for each study.

The patient-based pooled sensitivity and specificity values with 95% CI for BS using [^99m^Tc]Tc-diphosphonates were 90% (86–93; *I*^2^ = 83.2%) and 91% (87–94; *I*^2^ = 92.7%), respectively. Due to the different uptake mechanisms between [^18^F]FDG and Na[^18^F]F, the results related to their use in PET/CT have not been pooled together and were evaluated separately. The patient-based pooled sensitivity and specificity values with 95% CI for [^18^F]FDG PET/CT were 92% (88–95; *I*^2^ = 84.7%) and 99% (96–100; *I*^2^ = 44.6%), respectively. The patient-based pooled sensitivity and specificity values with 95% CI for Na[^18^F]F PET/CT were 96% (90–99; *I*^2^ = 55.6%) and 81% (72–88; *I*^2^ = 83.6%), respectively. The forest plots and SROC curves related to BS with [^99m^Tc]Tc-diphosphonates and [^18^F]FDG and Na[^18^F]F PET/CT are presented in Figure 4 and Figure 5.

The values of the area under the SROC curve for BS with [^99m^Tc]Tc-diphosphonates, [^18^F]FDG PET/CT, and Na[^18^F]F PET/CT were 0.93, 0.99, and 0.99, respectively.

For the different hybrid PET methods (PET/CT, PET/MRI), separate meta-analyses were not possible due to the limited number of studies.

### 3.5. Subgroup Analysis and Exploration of Heterogeneity

**Figure 6:**
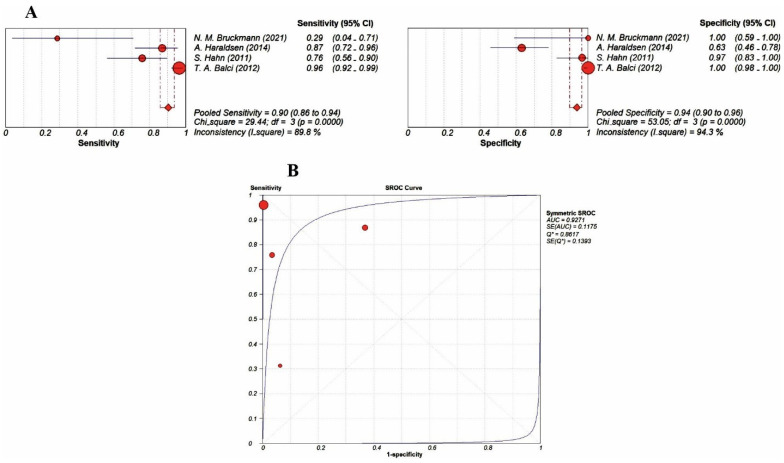
Forest plots (**A**) for the patient-based pooled sensitivity and specificity of planar BS with [^99m^Tc]Tc-diphosphonates [20,26,31,32] and SROC curve (**B**). The blue lines show the 95% confidence interval values and the red points show the values for each study. The dotted red lines indicate the range of pooled sensitivity and specificity values.

We performed a subgroup analysis to better evaluate the diagnostic performance of BS with [^99m^Tc]Tc-diphosphonates, taking into account the different type of acquisition (planar versus tomographic acquisition). Due to the limited number of studies using tomographic acquisition (SPECT), subgroup analysis was possible only for planar BS acquisition. The forest plots for patient-based pooled sensitivity and specificity and SROC curve for planar BS using [^99m^Tc]Tc-diphosphonates are presented in Figure 6. The value of the AUC was 0.93.

The values of pooled DORs and pooled LR^+^ and LR^−^ for each diagnostic method are shown in Table 4.

Furthermore, in the investigation of factors causing heterogeneity, estimation value details for some of the variables are listed in Table 5.

### 3.6. Analysis of Publication Bias

**Figure 7:**
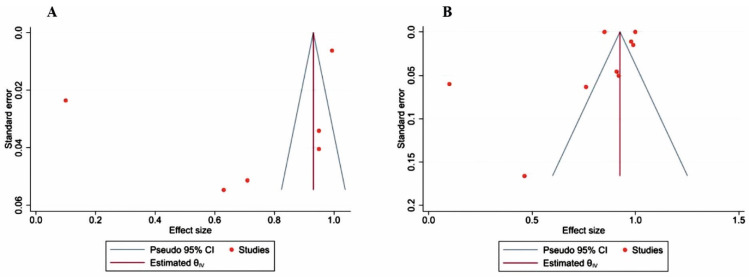
Funnel plots related to bone scan using [^99m^Tc]Tc-diphosphonates (**A**) and ([^18^F]FDG or Na[^18^F]F) PET/CT (**B**) for evaluation of publication bias. The specificity index was calculated as the effect size.

The result of the publication bias analysis is presented in Figure 7. Given the number of studies, the result should be considered with caution; however, taking into account the asymmetry of the graphs, possible publication bias cannot be excluded.

## 4. Discussion

The high risk of BMs in BrC patients causes a reduction in the life expectancy and quality of life of these patients. The diagnostic protocols for the detection of BMs in advanced BC patients usually include CT scanning and BS with [^99m^Tc]Tc-diphosphonates, and sometimes advanced BrC patients are referred to a [^18^F]FDG PET/CT for further investigation. However, with the development of medical equipment and hardware, diagnostic protocols need to be updated. The important matter is choosing the best nuclear medicine imaging method for timely treatment among BS and PET scans, taking into account that the cost-effectiveness ratio should be reasonable (because the cost of BS with [^99m^Tc]Tc-diphosphonates is less than half of that of PET and PET/CT) [33].

Detection of BMs in BrC by [^99m^Tc]Tc-diphosphonates BS or PET scan with both [^18^F]FDG and Na[^18^F]F is based on the increased bone metabolism and radiotracer uptake in the bone lesions. Whereas BS and PET are functional imaging methods, CT and MRI modalities may detect BMs of BrC due to their morphological changes. In recent years, combined systems of functional and morphological imaging modalities have resulted in the design of hybrid imaging equipment such as SPECT/CT for BS or PET/CT and PET/MRI, which are more accurate compared to single imaging modalities [33]. In particular, hybrid imaging modalities (PET/CT or PET/MRI) are the current gold standard for PET imaging.

We believe that the results of a comparative meta-analytic evaluation of BS with [^99m^Tc]Tc-diphosphonates and PET with several radiotracers (including the combined systems PET/CT and PET/MRI) obtained from the current study can help oncologists, nuclear medicine physicians, and radiologists in their knowledge of the best nuclear medicine imaging methods according to the gold standard for the diagnosis of BMs in BrC patients.

BMs in advanced BrC may have osteolytic, osteosclerotic, or mixed features. BS with [^99m^Tc]Tc-diphosphonates can detect bone osteoblastic activity at the site of BMs but can miss some of them [32]. Furthermore, BS with [^99m^Tc]Tc-diphosphonates has a suboptimal specificity as increased radiotracer uptake is possible in many other conditions associated with pathological bone turnover, including bone injuries, fractures, osteoarthritis, osteosclerosis, etc. Even if the specificity of BS could be increased by the use of the SPECT/CT method, the need for more specific imaging methods in the detection of BMs remains [34].

PET is an accurate nuclear medicine imaging method used in oncology, and unlike BS it can also detect metastatic involvement of BrC outside the skeleton. Na[^18^F]F and [^18^F]FDG are currently the most used PET radiotracers for detecting BMs in BrC [35]. As yet, no meta-analytic study has compared the diagnostic performance of [^18^F]FDG PET with Na[^18^F]F PET in detecting BMs in BrC.

After evaluating studies published from 2010 to June 2023, results from 753 patients with advanced BrC were included in our meta-analysis for comparison of the diagnostic accuracy of BS with [^99m^Tc]Tc-diphosphonates, [^18^F]FDG PET/CT, and Na[^18^F]F PET/CT.

BS with [^99m^Tc]Tc-diphosphonates was performed in the included studies using planar acquisitions and tomographic (SPECT or SPECT/CT) acquisitions in some cases. Traditional planar acquisition in both the anterior and posterior views is performed routinely compared to SPECT due to the reduced time required and better availability. The use of SPECT or SPECT/CT acquisitions reduces instances of overlapping lesions and missing metastases, especially in the spine and pelvic regions [36]. The main disadvantage of tomographic acquisitions compared to planar acquisitions is the additional time needed to obtain the images. Reconstruction algorithms with resolution recovery have been designed and presented to reduce SPECT scanning time, which can make BS with planar and SPECT acquisition a suitable option for bone metastases investigation [37]. Unfortunately, due to the limited number of studies, it was not possible to analyze BS with SPECT or SPECT/CT acquisition as a separate subgroup, so meta-analysis was performed only for the whole BS group and for the subgroup of planar acquisition. However, the values of pooled sensitivity (90%) and specificity (91%) of BS with [^99m^Tc]Tc-diphosphonates showed the good diagnostic accuracy of this imaging method in detecting BMs in BrC.

The pharmacokinetic properties and uptake mechanisms of [^18^F]FDG and Na[^18^F]F are different. [^18^F]FDG usually accumulates at the site of active and living tumor cells due to their increased glucose metabolism, whereas Na[^18^F]F accumulates in bone crystals, and its uptake increases in cases of increased bone turnover [38,39,40,41]. According to our analysis, [^18^F]FDG PET was more accurate in detecting osteolytic lesions compared to [^99m^Tc]Tc-diphosphonates BS, even if performed with SPECT acquisition [33], with higher sensitivity and specificity, though it can miss some osteosclerotic lesions [42,43]. Na[^18^F]F PET/CT showed excellent sensitivity in detecting BMs in BrC, but lower specificity compared to [^18^F]FDG PET/CT and BS, and poor evaluation of metastatic sites outside the skeleton compared to [^18^F]FDG PET/CT.

An interesting point is that in patients undergoing bisphosphonate therapy, uptake of Na[^18^F]F increased whereas [^18^F]FDG uptake decreased. This different uptake likely indicates the effectiveness of the treatment, reduction of living cells in the tumor, bone mineralization, and sclerosis. On the other hand, some researchers believe that the reduction of Na[^18^F]F uptake in follow-up images can be evidence of recovery and normal metabolic activity, which confirms successful treatment [30,44].

Most bone metastases in BrC are detected in the spine, pelvic regions, and ribs (the number of true positive lesions were more frequent in the pelvic region); this indicates the need for an accurate diagnostic tool to evaluate these areas. In this regard, [^18^F]FDG PET was more accurate in detecting metastases in the spine and Na[^18^F]F PET was more accurate in detecting metastases in the ribs. This difference may be related to structural changes caused by metastases as a function of their anatomical localization.

Delayed acquisition of [^18^F]FDG PET (at about 2 h after radiotracer injection) to differentiate between benign and malignant lesions did not affect the sensitivity and only provided a better lesion-to-background contrast [21].

Some limitations of the studies included in the meta-analysis should be underlined: the patient follow-up for all studies was mostly based on imaging, and histopathology was not used as a reference standard in most of the studies. On the other hand, histopathology sampling of all the suspicious metastatic lesions at imaging seems to be infeasible and unnecessary.

A limitation of our meta-analysis was the significant number of retrospective studies included, as retrospective studies are affected by some biases due to patient selection. Furthermore, we should recognize that little information was available on the use of some multimodal imaging methods such as SPECT/CT and PET/MRI (which is currently less available than PET/CT worldwide) for detecting BMs in BrC [45]. In particular, SPECT/CT with radiolabeled diphosphonates is a powerful diagnostic imaging method in the diagnosis of BMs [46]. Quantitative SPECT/CT with radiolabeled diphosphonates could be useful to improve the detection of BMs and to facilitate the differential diagnosis of BMs and benign bone lesions, but confirmatory prospective multicenter studies on the usefulness of quantitative SPECT/CT in this setting are needed [47].

Other limitations of our meta-analysis include the significant statistical heterogeneity and possible publication bias. We have explored the statistical heterogeneity and believe that several factors including patients’ characteristics, index test characteristics, and methodological aspects of the included studies may explain this heterogeneity.

We did not perform a cost-effectiveness analysis, but we have focused our analysis only on the diagnostic accuracy of different nuclear medicine and multimodal imaging methods in detecting BMs in BrC. BS is a low-cost and more available method compared to PET/CT and PET/MRI. However, the higher diagnostic accuracy of [^18^F]FDG PET and related hybrid modalities compared to BS in detecting BMs in BrC, the advantage of [^18^F]FDG PET and related hybrid modalities in evaluating metastatic sites outside the skeleton compared to BS and Na[^18^F]F PET, and the higher availability of PET/CT compared to PET/MRI, suggest that [^18^F]FDG PET/CT should be the preferred multimodal imaging method to evaluate BMs in BrC, if available. We believe that this evidence-based information could be used to update the existing guidelines on advanced BrC imaging.

Multimodal medical image segmentation is a crucial task that enables the precise localization and quantification of tumors. In the context of hybrid imaging and multimodal PET/CT and PET/MRI segmentation, automated approaches for segmentation could assist the diagnostic performance of PET/CT and PET/MRI with different radiotracers for detecting bone metastases in BrC [48,49,50].

## 5. Conclusions

BS has good diagnostic performance in detecting BMs in BrC. However, due to its higher and balanced sensitivity and specificity, and its advantage in evaluating extra-skeletal lesions, [^18^F]FDG PET(/CT) should be the preferred multimodal imaging method for the evaluation of BMs in BrC, if available.

## Figures and Tables

**Table 1:** Descriptive specifications of basic studies and patients’ characteristics included in the systematic evaluation.

First Author/Year of Publication	Country	Sample Size	Age Mean (Min–Max) in Years	Study Type	Test Type	PET Device Model	BS Device Model
Bruckmann et al. [20]/2021	Germany	154	NR	Prospective	[^18^F]FDG PET/MRI [^99m^Tc]Tc-HDP BS (Planar)	NR	Symbia S, Siemens Healthineers, Germany
Hansen et al. [21]/2021	Denmark	18	61.5 (38–76)	Prospective	[^18^F]FDG PET/CT [^99m^Tc]Tc-DPD BS (Planar)	Philips Medical, Surrey, UK	GE Healthcare Systems, Chicago, IL, USA
Abd-Elkader et al. [22]/2020	Egypt	71	54.7 (30–79)	Retrospective	[^18^F]FDG PET [^18^F]FDG PET/CT	Gemini, Philips Medical Systems, Netherlands	-
Rager et al. [23]/2018	Switzerland	25	NR	Retrospective	[^18^F]FDG PET/CT	Biograph 16-slice PET/CT scanner, Siemens Healthcare, Erlangen, Germany	-
Capitanio et al. [24]/2016	Italy	45	61	Prospective	[^18^F]FDG PET/CT Na[^18^F]F PET/CT	(1) Siemens Medical Solutions, Knoxville TN, USA (2) GE Medical Systems, Milwaukee, WI, USA	-
Abikhzer et al. [25]/2016	Israel	41	58 (30–75)	Prospective	Na[^18^F]F PET/CT [^99m^Tc]Tc-MDP BS (SPECT)	GE Healthcare, Waukesha, Wisconsin, USA	GE Healthcare, Waukesha, Wisconsin, USA
Haraldsen et al. [26]/2014	Denmark	76	61.7 (29–89)	Prospective	[^99m^Tc]Tc-DPD BS (Planar) [^99m^Tc]Tc-DPD BS (SPECT)	-	Philips Healthcare, Eindhoven, Netherlands
Caglar et al. [27]/2015	Turkey	150	52 (27–85)	Retrospective	[^18^F]FDG PET/CT [^99m^Tc]Tc-MDP BS (Planar)	GE Medical Systems, Waukesha, WI, USA	Infinia GP3, GE Healthcare, Milwaukee, WI, USA
Catalano et al. [28]/2015	Italy	109	58.1	Retrospective	[^18^F]FDG PET/CT [^18^F]FDG PET/MRI	Gemini TF; Philips, Best, Netherlands Siemens Healthcare, Erlangen, Germany	-
Yoon et al. [29]/2013	Korea	9	55.6	Prospective	Na[^18^F]F PET/CT	GE Healthcare, USA	-
Piccardo et al. [30]/2012	Italy	32	60	Retrospective	Na[^18^F]F PET/CT	General Electric Medical Systems, Milwaukee, WI, USA	-
Balci et al. [31]/2012	Turkey	162	50.6	Retrospective	[^18^F]FDG PET/CT [^99m^Tc]Tc-MDP BS (Planar)	Siemens AG, Munich, Germany	GE Healthcare Israel Ltd., Tirat Hacarmel, Israel
Hahn et al. [32]/2011	Germany	29	58 (35–78)	Retrospective	[^18^F]FDG PET/CT [^99m^Tc]Tc-MDP BS (Planar)	Siemens Molecular Imaging, Hoffman Estates, IL, USA	Symbia S Siemens, Erlangen, Germany

NR = not reported, BS = bone scintigraphy, PET = positron emission tomography; SPECT = Single Photon Emission Computed Tomography.

**Table 2:** Index test characteristics of studies included in the systematic evaluation.

First Author/Year of Publication	Test Type	Injected Activity (MBq or MBq/kg)	Waiting Time for Imaging	Bed Positions	Time per Bed Position	Most Frequent Bone Metastases	Follow-Up Time	Most Frequent Sites of Bone Metastases
Bruckmann et al. [20]/2021	[^18^F]FDG PET/MRI	254.4 MBq	64 min	3–5	3 min	Osteolytic	3.8 ± 1.3 months	Vertebrae > Pelvic > Limbs > Ribs
	[^99m^Tc]Tc-HDP BS	700 MBq	20–35 min	-	-	Osteolytic		
Hansen et al. [21]/2021	[^18^F]FDG PET/CT	4 MBq/kg	210 min	7–9	(2.5–3.5) min	Osteolytic	NR	NR
	[^99m^Tc]Tc-DPD BS	700 MBq	240 min	-	-	Osteolytic		
Abd-Elkader et al. [22]/2020	[^18^F]FDG PET	2.43–4.59 MBq/kg	45 min	8	2.5 min	Osteolytic	NR	NR
	[^18^F]FDG PET/CT	2.43–4.59 MBq/kg	45 min	8	2.5 min	Osteolytic		
Rager et al. [23]/2018	[^18^F]FDG PET/CT	370 MBq	60 min	7–9	3 min	NR	21 months	Vertebrae > Ribs > Pelvic > Sacrum > Humerus > Clavicle, Sternum > Cranium, Femur > Clavicle
Capitanio et al. [24]/2016	[^18^F]FDG PET/CT	NR	NR	NR	NR	Osteolytic	12 months	Ribs > Spine
	Na[^18^F]F PET/CT	NR	NR	NR	NR	Osteosclerotic		
Abikhzer et al. [25]/2016	Na[^18^F]F PET/CT	333–555 MBq	60 min	9	2.5 min	NR	33 months	Pelvic > Thoracic > Lumbar spine > Ribs > Cervical
	[^99m^Tc]Tc-MDP BS (SPECT)	925 MBq	120 min	-	-	NR		
Haraldsen et al. [26]/2014	[^99m^Tc]Tc-DPD BS (Planar)	750 MBq	180 min	-	-	NR	NR	NR
	[^99m^Tc]Tc-DPD BS (SPECT)	750 MBq	180 min	-	-	NR		
Caglar et al. [27]/2015	[^18^F]FDG PET/CT	259–370 MBq	60 min	NR	3 min	NR	22 months	Vertebrae > Pelvic > Ribs > Sternum, Clavicles, Scapula > Extremities > Skull
	[^99m^Tc]Tc-MDP BS (Planar)	740 MBq	NR	-	-	NR		
Catalano et al. [28]/2015	[^18^F]FDG PET/CT	370–400 MBq	60 min	5–7	1.5 min	Osteolytic	347–621 days	Pelvic > Vertebrae > Sternum > Ribs > Appendicular
	[^18^F]FDG PET/MRI	370–400 MBq	120 min	5–6	4 min	Osteolytic		
Yoon et al. [29]/2013	Na[^18^F]F PET/CT	370 MBq	60 min	7–8	3 min	Osteosclerotic	14.3 ± 7.6 months	Vertebrae > Pelvic > Thoracic > Extremities
Piccardo et al. [30]/2012	Na[^18^F]F PET/CT	370 MBq	60 min	10–12	3 min	NR	12 months	Spine > Thorax > Pelvic > Extremities > Skull
Balci et al. [31]/ 2012	[^18^F]FDG PET/CT	370–550 MBq	60 min	NR	NR	NR	NR	NR
	[^99m^Tc]Tc-MDP BS (Planar)	550–1000 MBq	120–180 min	-	-	NR		
Hahn et al. [32]/2011	[^18^F]FDG PET/CT	184–340 MBq	60 min	NR	NR	Osteolytic	170–425 days	NR
	[^99m^Tc]Tc-MDP BS (Planar)	NR	NR	-	-	Osteolytic		

NR = not reported, BS = bone scintigraphy, PET = positron emission tomography; SPECT = Single Photon Emission Computed Tomography.

**Table 3:** Reference standard and diagnostic results of index test and comparator for all the 11 studies included in the meta-analysis.

First Author	Reference Standard	Test Type	Per Patient				Per Lesion			
			TP	FP	FN	TN	TP	FP	FN	TN
Bruckmann et al. [20]	CT or MRI scan	[^18^F]FDG PET/MRI [^99m^Tc]Tc-HDP BS (Planar)	7 2	0 1	0 5	7 6	41 15	0 -	0 28	41 -
Abd-Elkader et al. [22]	MRI, and bone scan	[^18^F]FDG-PET [^18^F]FDG PET/CT	9 71	62 0	0 0	0 71	1 6	65 65	0 0	5 0
Rager et al. [23]	CT or MRI scan (PET, SPECT/CT, bone scan in some cases)	[^18^F]FDG PET/CT	10	0	2	13	43	0	48	18
Capitanio et al. [24]	Physical examination, blood tests, CT, MRI, [^18^F]FDG-PET/CT, X-ray, and bone scan	[^18^F]FDG PET/CT Na[^18^F]F PET/CT	34 41	1 11	11 4	44 34	160 219	9 202	84 25	235 42
Abikhzer et al. [25]	CT	Na[^18^F]F PET/CT [^99m^Tc]Tc-MDP BS (SPECT)	21 19	3 1	0 2	17 19	76 50	9 6	195 198	4 30
Haraldsen et al. [26]	MRI	[^99m^Tc]Tc-DPD BS (Planar) [^99m^Tc]Tc-DPD BS (SPECT)	33 33	14 11	5 5	24 27	- -	- -	- -	- -
Catalano et al. [28]	Bone scan, PET/CT, and PET/MRI	[^18^F]FDG PET/MRI	24	0	1	25	135	3	6	138
Piccardo et al. [30]	Physical examination, blood tests, CT, MRI, [^18^F]FDG-PET/CT, X-ray, and bone scan	Na[^18^F]F PET/CT	27	0	0	27	-	-	-	-
Yoon et al. [29]	Blood tests and imaging	Na[^18^F]F PET/CT	9	5	0	4	49	36	3	31
Balci et al. [31]	[^18^F]FDG PET/CT and bone scan	[^18^F]FDG PET/CT [^99m^Tc]Tc-MDP BS (Planar)	47 40	0 0	2 9	49 49	- -	- -	- -	- -
Hahn et al. [32]	[^18^F]FDG PET/CT, MRI, and bone scan	[^18^F]FDG PET/CT [^99m^Tc]Tc-MDP BS (Planar)	28 22	2 1	1 7	27 28	67 53	54 56	3 17	5 3

True Positive (TP), False Positive (FP), False Negative (FN), and True Negative (TN).

**Table 4:** The values of diagnostic odds ratios (DORs) and likelihood ratios (LRs) for BS, [^18^F]FDG PET(/CT), and Na[^18^F]F PET(/CT) and related 95% confidence interval values calculated using a random-effects method.

Test Type	LR		DOR
	LR^+^	LR^−^	
[^99m^Tc]Tc-diphosphonates bone scan	10.2 (2.2–47.9)	0.18 (0.06–0.56)	62.6 (10.4–375)
[^99m^Tc]Tc-diphosphonates bone scan (planar only)	16.2 (0.2–1095)	0.20 (0.04–1.02)	80.3 (3.8–1707)
[^18^F]FDG PET/CT	28.2 (11.4–69.9)	0.08 (0.02–0.29)	510.7 (102.5–2547)
Na[^18^F]F PET/CT	4.2 (1.6–11.3)	0.09 (0.04–0.20)	84.7(12.7–564)

LR: likelihood ratio, DOR: diagnostic odds ratio.

**Table 5:** Details of the test to investigate factors affecting heterogeneity in [^99m^Tc]Tc-diphosphonates bone scan and ([^18^F]FDG-Na[^18^F]F) PET/CT.

Variables	[^99m^Tc]Tc-Diphosphonates BS				([^18^F]FDG-Na[^18^F]F) PET/CT			
	Coefficient	SD	Z	P > Z	Coefficient	SD	Z	P > Z
**Publication year**	0.0060353	0.021	0.28	0.777	0	0.011	0.00	1.00
**Sample size**	0.0012897	0.001	0.97	0.331	0	0.001	0.00	1.00
**Type of study**	−0.110445	0.143	−0.77	0.442	0	0.046	0.00	1.00

## Data Availability

Not applicable.

## Supplementary Materials

The following supporting information can be downloaded at: https://www.mdpi.com/article/10.3390/jimaging9120274/s1.
